# Atrial fibrillation cryoablation complicated by diffuse coronary artery vasospasm and cardiac arrest: a case report

**DOI:** 10.1093/ehjcr/ytag649

**Published:** 2026-08-31

**Authors:** Casey L Nash, Zhongrui Zhou, James Clark, Vivekananda Joshi, Andrei D Margulescu

**Affiliations:** Morriston Cardiac Centre, Morriston Hospital, Swansea Bay University Health Board, Heol Maes Egwlys, Swansea SA6 6NL, UK; Morriston Cardiac Centre, Morriston Hospital, Swansea Bay University Health Board, Heol Maes Egwlys, Swansea SA6 6NL, UK; Morriston Cardiac Centre, Morriston Hospital, Swansea Bay University Health Board, Heol Maes Egwlys, Swansea SA6 6NL, UK; Department of Cardiology, Royal Glamorgan Hospital, Cwm Taf Morgannwg University Health Board, Ynysmaerdy Terrace, Pontyclun CF72 8XR, UK; Morriston Cardiac Centre, Morriston Hospital, Swansea Bay University Health Board, Heol Maes Egwlys, Swansea SA6 6NL, UK; Morriston Cardiac Centre, Morriston Hospital, Swansea Bay University Health Board, Heol Maes Egwlys, Swansea SA6 6NL, UK

**Keywords:** Atrial fibrillation, Coronary artery spasm, Cryoballoon ablation, ST-segment elevation, Ventricular fibrillation, Case report

## Abstract

**Background:**

Pulmonary vein isolation (PVI) is recommended for rhythm control in selected patients with symptomatic atrial fibrillation (AF). Coronary artery spasm (CAS) is a rare but recognized complication of AF ablation and may result in life-threatening arrhythmias.

**Case summary:**

A 47-year-old man underwent cryoballoon PVI for symptomatic paroxysmal AF. Transient inferior ST-segment elevation occurred following left-sided PVI and resolved spontaneously. Shortly after completion of the procedure, recurrent ST-segment elevation developed, progressed to malignant ventricular arrhythmia and cardiac arrest, and emergency angiography demonstrated severe diffuse spasm affecting both coronary systems without obstructive disease. Return of spontaneous circulation occurred following spontaneous resolution of CAS before intracoronary nitrate administration. Ventricular function normalized within 24 h. Recovery was complicated by transient right phrenic nerve palsy and persistent post-traumatic stress disorder. At 2-year follow-up, there was no recurrent AF on implantable loop recorder monitoring.

**Discussion:**

This case highlights delayed, recurrent and diffuse CAS as a rare but potentially catastrophic complication of cryoballoon AF ablation. Continuous electrocardiographic monitoring in the peri-procedural period is essential. Although spontaneous resolution occurred in this case, previous reports support rapid access to intravenous or intracoronary nitrates. Emerging data from pulsed field ablation suggests that coronary vasospasm is not restricted to cryothermal energy and that nitroglycerine prophylaxis may reduce, but not abolish, risk.

Learning points

- Transient ST-segment elevation during or after pulmonary vein isolation may be an early warning sign of evolving coronary vasospasm.
- Coronary vasospasm may be delayed, recurrent and diffuse, and may progress rapidly to malignant ventricular arrhythmia and cardiac arrest.
- Coronary vasospasm is increasingly recognized across ablation energy sources; nitroglycerine prophylaxis may reduce severe vasospasm in selected settings but does not eliminate risk.

## Introduction

Catheter ablation with pulmonary vein isolation (PVI) is an established rhythm-control therapy for selected patients with symptomatic atrial fibrillation (AF).^1^ Although the procedure is generally safe, rare but potentially life-threatening complications may occur.^2^ Coronary artery spasm (CAS) is an uncommon complication of AF ablation that has been reported with radiofrequency, cryoballoon and pulsed field ablation (PFA). Among thermal techniques, CAS has been reported more frequently following cryoballoon than radiofrequency ablation, while large contemporary PFA registries have reported an incidence of ∼1 per 1000 procedures.^3–8^ Coronary artery spasm may result in myocardial ischaemia, malignant ventricular arrhythmias and cardiac arrest, requiring prompt recognition and management.^3–8^

## Summary figure

**Figure:**
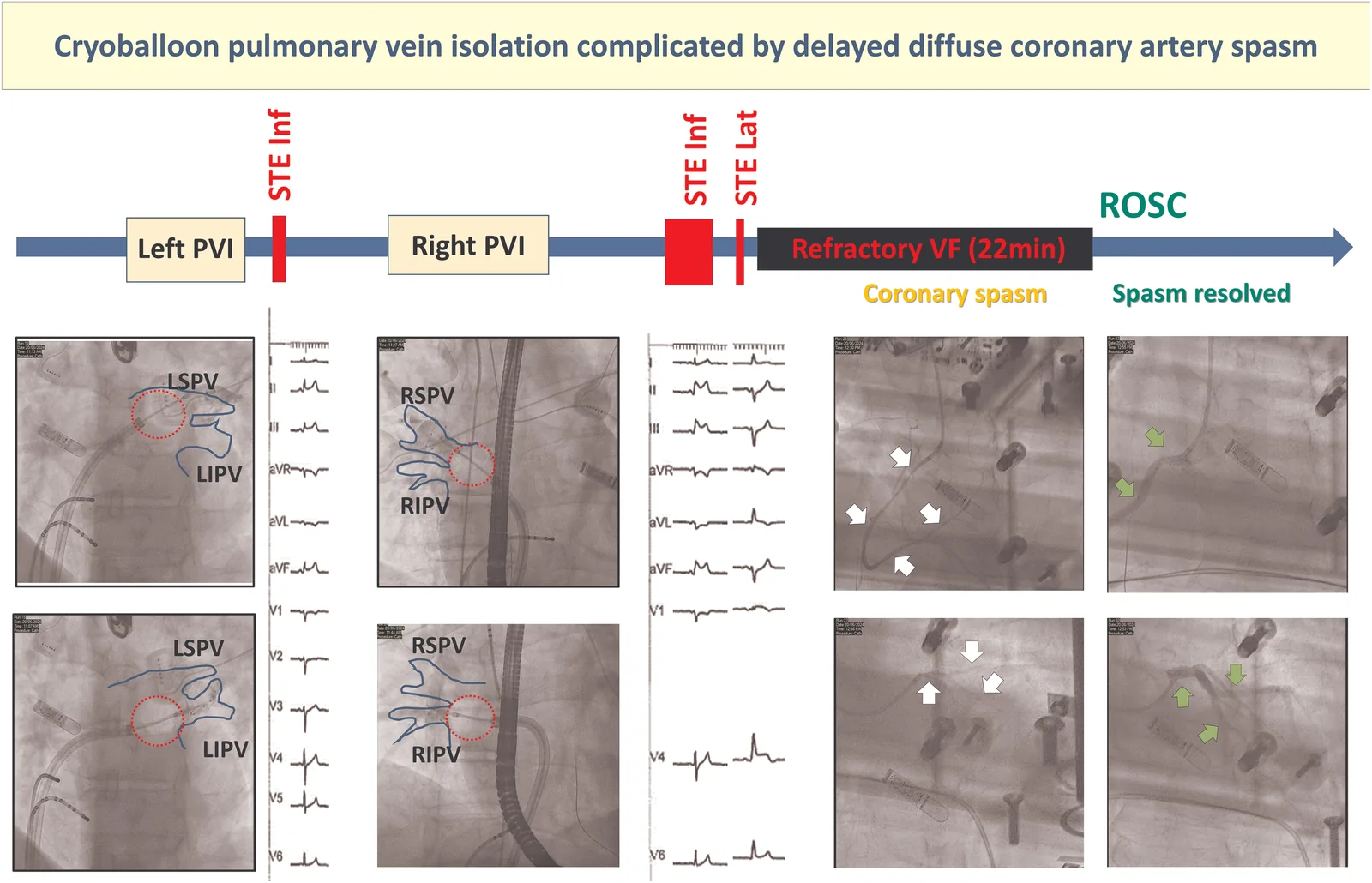
Graphical summary of the case, showing the sequence from cryoballoon pulmonary vein isolation to recurrent ST-segment elevation, diffuse bilateral coronary artery spasm (white arrows), ventricular arrhythmia/cardiac arrest, spontaneous resolution of coronary spasm (green arrows), and recovery. *Alternative text:* Multipanel graphical summary combining a procedural timeline, representative ECG changes and coronary angiography images, illustrating delayed recurrent coronary spasm and cardiac arrest after cryoballoon ablation. PVI, pulmonary vein isolation; STE, ST segment elevation; Inf, inferior ECG leads; Lat, lateral ECG leads; LSPV, left superior pulmonary vein; LIPV, left inferior pulmonary vein; RSPV, right superior pulmonary vein; RIPV, right inferior pulmonary vein; VF, ventricular fibrillation; ROSC, return of spontaneous circulation.

## Case presentation

A 47-year-old man with hypertension, increased alcohol intake and a 1-year history of highly symptomatic paroxysmal AF refractory to flecainide and beta-blockers was referred for catheter ablation. He was anticoagulated with apixaban. AF episodes were documented on an implantable loop recorder (LinQ, Medtronic, USA). Baseline echocardiography demonstrated mild left ventricular hypertrophy with preserved left ventricular ejection fraction and no structural abnormalities.

Cryoballoon PVI was performed under uninterrupted anticoagulation. The procedure was performed under general anaesthesia. General anaesthesia was induced with propofol and remifentanil target-controlled infusion and the airway was secured with an endotracheal tube. Processed electroencephalography monitoring (SedLine, Masimo, Irvine, CA, USA) was used to titrate anaesthetic depth. Right femoral venous access was achieved under ultrasound guidance. Transseptal puncture was guided by transoesophageal echocardiography, and adequate anticoagulation with activated clotting time >350 s was maintained using unfractionated Heparin. A cryoballoon system (Arctic Front Advance, Medtronic, USA) was used.

A left common pulmonary vein trunk was identified and successful left-sided PVI was achieved (*Figure 1*). Approximately 1 min after completion of left PVI, transient ST-segment elevation was observed in the inferior electrocardiogram (ECG) leads, resolving spontaneously within 3 min without haemodynamic compromise (*Figure 2A*). Right-sided PVI was subsequently completed with phrenic nerve pacing, achieving complete isolation.

**Figure 1:**
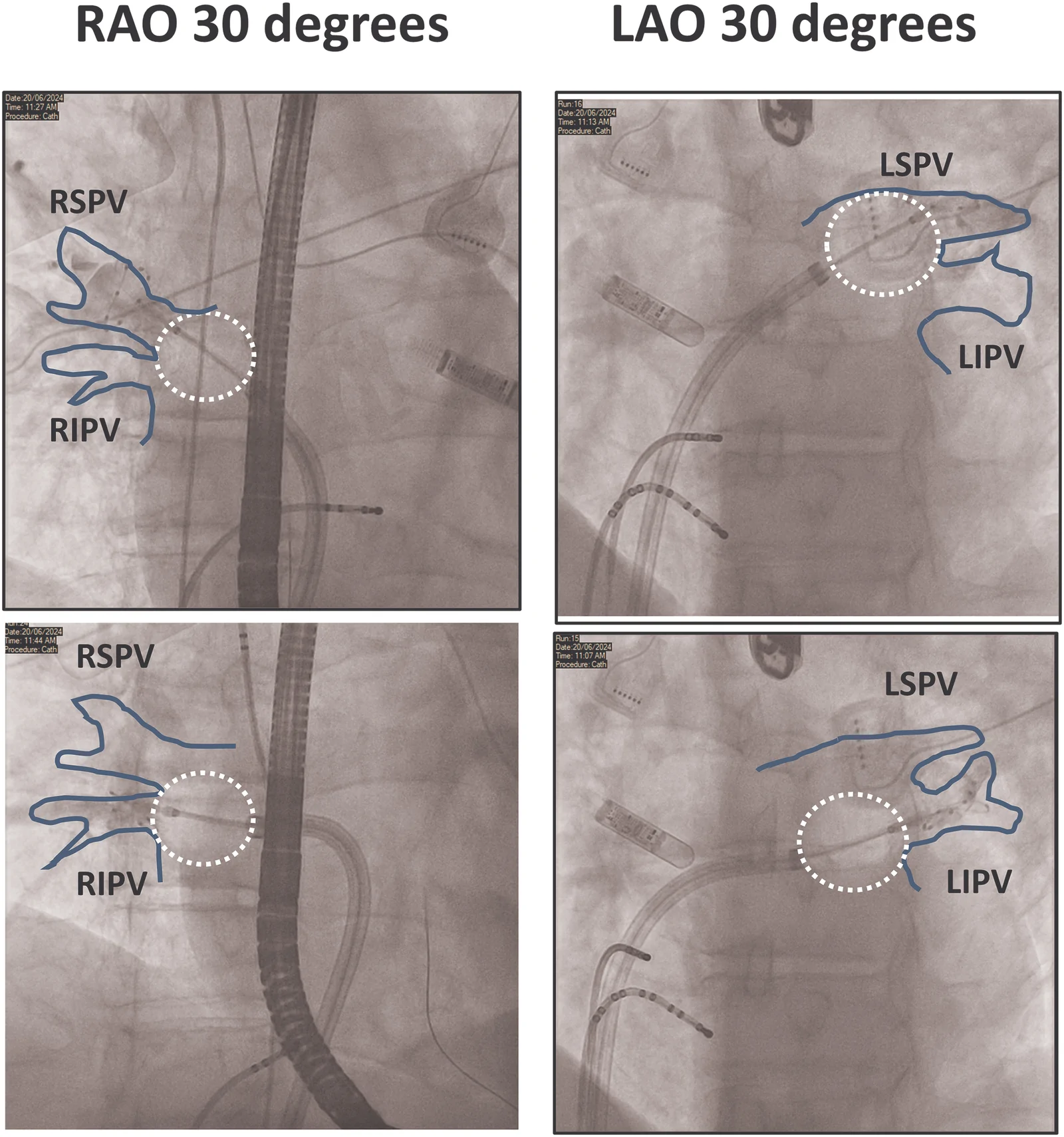
Cryoballoon pulmonary vein isolation. Position of the cryoballoon ablation catheter (interrupted circles) in relation to the four pulmonary vein ostia (outlined) on fluoroscopic views (right anterior oblique, RAO, 30 degrees; left anterior oblique, LAO, 30 degrees). LSPV, left superior pulmonary vein; LIPV, left inferior pulmonary vein; RSPV, right superior pulmonary vein; RIPV, right inferior pulmonary vein; RAO, right anterior oblique view; LAO, left anterior oblique view.

**Figure 2:**
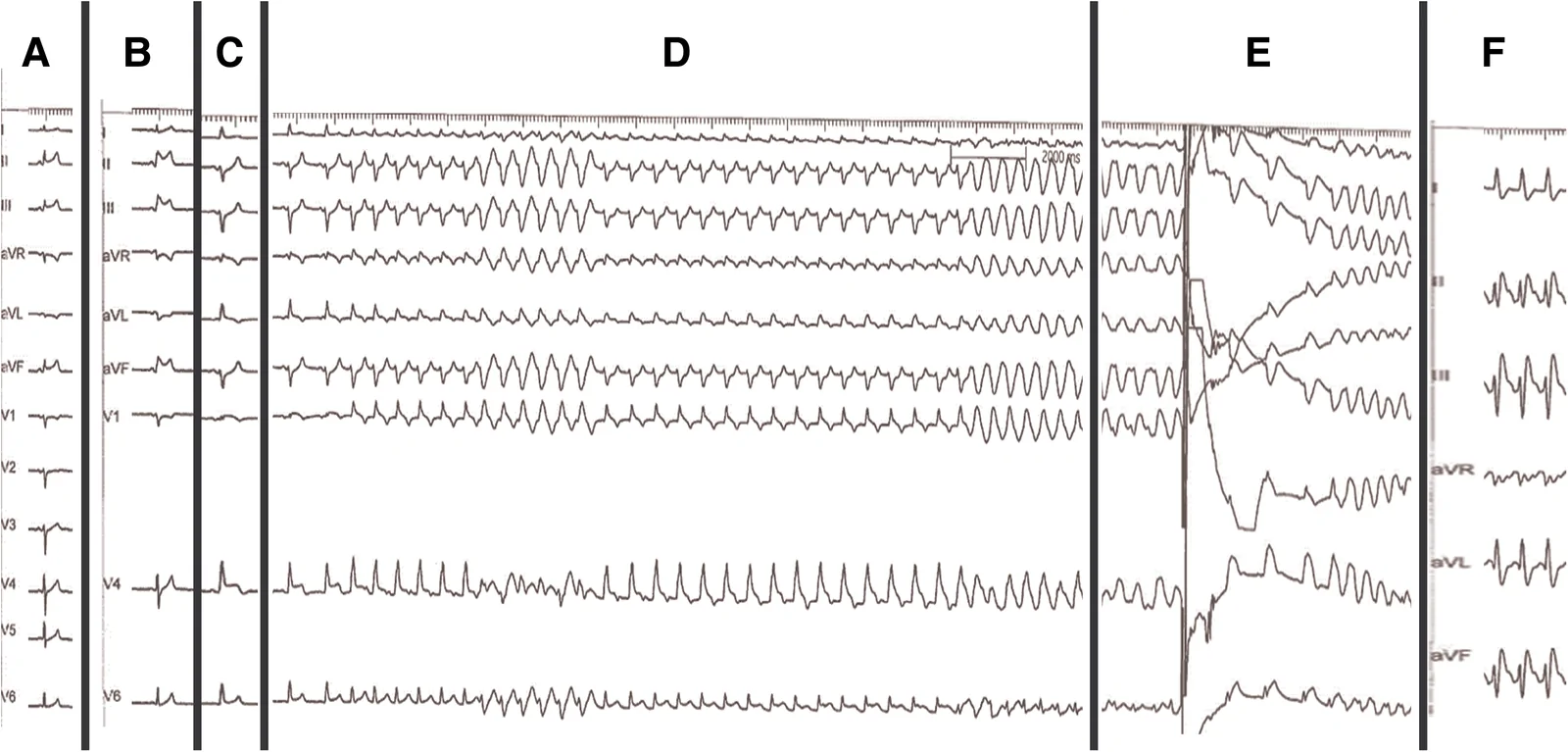
Electrocardiogram sequence during recurrent ST-segment elevation and cardiac arrest. (*A*) Inferior ST-segment elevation ∼1 min after left pulmonary vein isolation, resolving within 3 min. (*B*) Recurrent inferior ST-segment elevation ∼13 min after final ablation, resolving after 5 min. (*C*) Lateral ST-segment elevation. (*D*) Progression to idioventricular rhythm, ventricular tachycardia and coarse ventricular fibrillation. (*E*) Unsuccessful electrical cardioversion attempt. (*F*) Return of spontaneous circulation before intracoronary nitrate administration.

Approximately 13 min after the final ablation, ST-segment elevation recurred in the inferior leads and resolved spontaneously after 5 min (*Figure 2B*). A further episode shortly afterwards involved the lateral leads (*Figure 2C*) and progressed rapidly to idioventricular rhythm, polymorphic ventricular tachycardia and coarse ventricular fibrillation (*Figure 2D*).

Emergency coronary angiography demonstrated severe diffuse spasm affecting both right and left coronary arteries, without obstructive disease (*Figure 3A* and *B*; *Videos 1* and *2*). Advanced life support was initiated, including multiple defibrillation attempts, adrenaline and amiodarone. Chest compressions were performed manually and subsequently using a LUCAS mechanical compression device (Stryker). Before intracoronary nitrate administration, spontaneous resolution of CAS occurred, with return of spontaneous circulation after ∼22 min (*Figure 2E* and *F*). Repeat angiography confirmed complete resolution of spasm (*Figure 3C* and *D*; *Videos 3* and *4*). The full time sequence of events is displayed in *Figure 4*.

**Figure 3:**
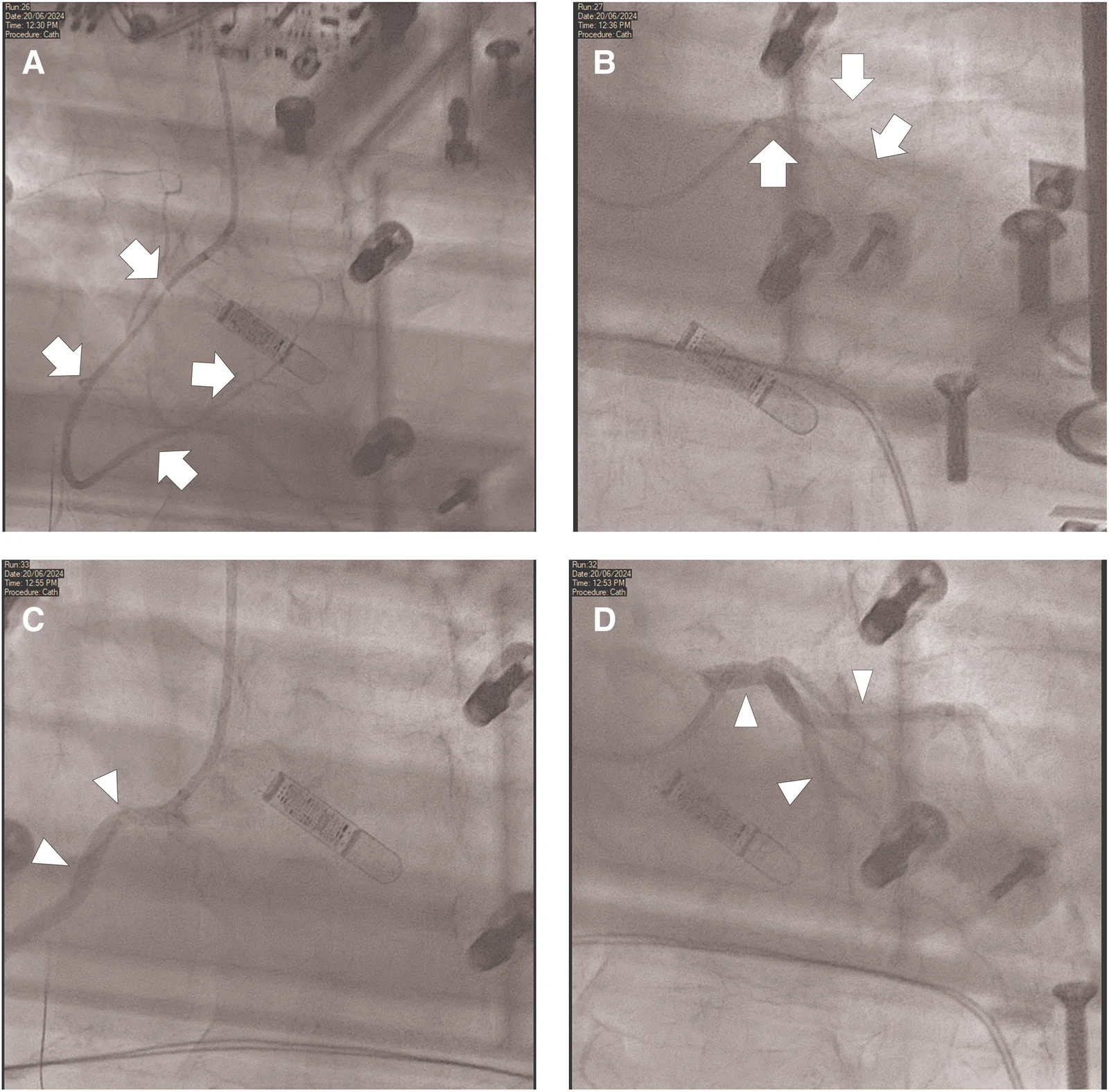
Emergency coronary angiography. Emergency coronary angiography during cardiopulmonary resuscitation demonstrated diffuse severe spasm of the right coronary artery (*A*) and left coronary system including the left main stem (*B*) (arrows), with complete spontaneous resolution on repeat angiography (*C*, right coronary artery; *D*, left coronary system) (arrowheads).

**Figure 4:**
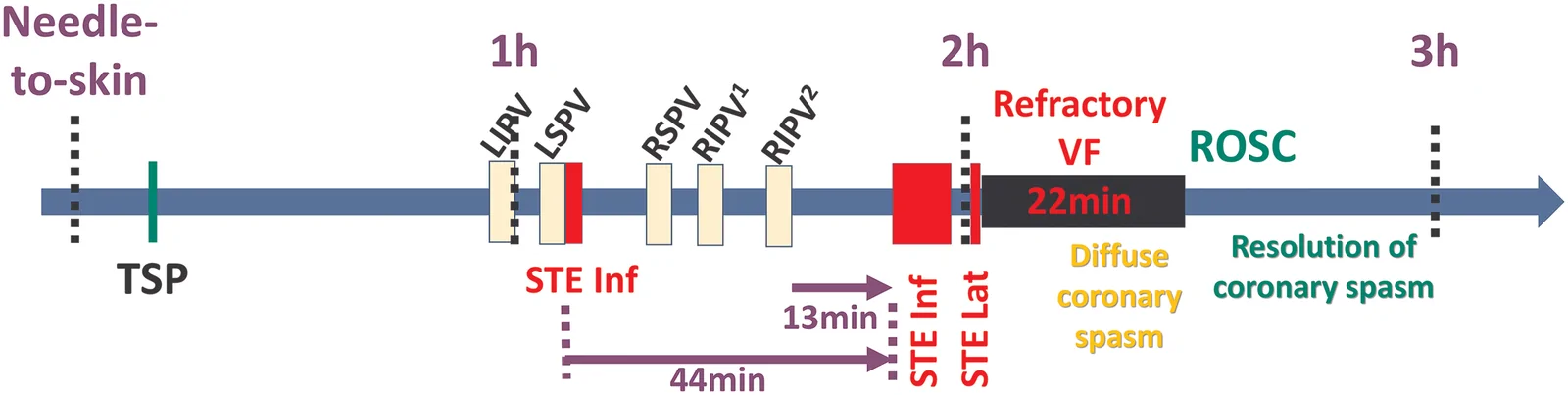
Time sequence of events. Rectangles labelled with individual pulmonary vein abbreviations depict the timing and duration of cryoballoon ablation of each pulmonary vein. Rectangles labelled ‘STE' depict episodes of ST-segment elevation, and the rectangle labelled ‘Refractory VF’ depicts cardiac arrest. Double-headed arrows above the timeline indicate the elapsed time intervals between the events marked by their endpoints. The timing of coronary angiography, resolution of coronary spasm, and return of spontaneous circulation are also displayed. Inf, inferior ECG leads; Lat, lateral ECG leads; LIPV, left inferior pulmonary vein; LSPV, left superior pulmonary vein; ROSC, return of spontaneous circulation; RIPV, right inferior pulmonary vein; RSPV, right superior pulmonary vein; STE, ST-segment elevation; TSP, transseptal puncture; VF, ventricular fibrillation.

Post-resuscitation echocardiography demonstrated transient right coronary territory hypokinesia with left ventricular ejection fraction of 40%, which normalized within 24 h. The patient required short-term vasopressor support and was weaned from mechanical ventilation within 24 h of Intensive Care Unit admission. Recovery was complicated by chest trauma related to resuscitation and transient right phrenic nerve palsy, both resolving by 6 months. The patient developed persistent post-traumatic stress disorder. Long-term rhythm monitoring revealed only one AF episode during early recovery and no subsequent recurrences at 2-year follow-up.

## Discussion

Coronary artery spasm is a rare but clinically significant complication of AF ablation and has been reported with radiofrequency, cryoballoon and PFA. In a large multicentre series predominantly reflecting thermal ablation practice, the overall incidence was 0.19%, with a higher reported incidence following cryoballoon than radiofrequency ablation (0.33% vs. 0.04%).^3,4^ Large contemporary PFA registries have reported clinically recognized CAS in ∼0.06%–0.10% of procedures.^7,8^ Although uncommon, CAS may cause myocardial ischaemia, malignant ventricular arrhythmias and cardiac arrest, as occurred in this case.^3,4,7–9^

The underlying mechanism is likely multifactorial and may vary according to the energy source, anatomical site and procedural context. Autonomic imbalance is a leading hypothesis: ablation near the pulmonary veins, particularly the left-sided veins, may affect adjacent ganglionated plexi and provoke coronary vasomotor instability.^4^ Coronary artery spasm has also occurred during procedural steps associated with autonomic stimulation, including transseptal puncture and catheter manipulation.^3,4^ Endothelial dysfunction and coronary hyperreactivity may increase susceptibility, particularly in patients with risk factors such as smoking and hypertension.^4^ In cryoballoon ablation, cooling of circulating blood or adjacent tissues has additionally been proposed, although a direct cryothermal mechanism alone does not readily explain delayed, recurrent or anatomically remote spasm.^5,6^

Pulsed field ablation has renewed attention to ablation-related CAS. In addition to clinically recognized events during PVI, delayed myocardial ischaemia, ventricular arrhythmias and sudden cardiac death following PFA have recently been described.^7–9^ When PFA is delivered close to a coronary artery, particularly during cavotricuspid or mitral isthmus ablation, transient angiographic spasm may occur considerably more frequently than clinically apparent events reported in PVI registries.^10,11^ High-dose parenteral nitroglycerine prophylaxis reduces the severity and frequency of PFA-related vasospasm but does not eliminate either angiographic spasm or clinically significant events; CAS despite prophylaxis has been reported.^10,12,13^

Clinically, CAS may occur during or after ablation.^3,14^ Transient ST-segment elevation, most often in the inferior leads, may be the earliest sign and may precede haemodynamic compromise. In the present case, recurrent inferior ST-segment elevation before arrest was an important warning sign. Air embolism remains an important differential diagnosis during left atrial procedures.^14^ However, recurrent episodes over time, diffuse bilateral coronary narrowing and complete resolution favoured vasospasm rather than fixed embolic obstruction.

Management is centred on rapid coronary vasodilatation. Intravenous or intracoronary nitrates are the mainstay of treatment and have been associated with prompt spasm resolution.^3,10^ Although CAS resolved spontaneously before nitrate administration in this case, immediate availability of nitrates remains essential. Transient right phrenic nerve palsy was also observed in our case. This is a recognized cryoballoon complication due to the close anatomical relationship between the right phrenic nerve and right-sided pulmonary veins.^5^

## Conclusion

Diffuse CAS is a rare but potentially life-threatening complication of cryoballoon AF ablation. Continuous ECG monitoring and early recognition of ST-segment changes are essential. Experience from both thermal and PFA supports vigilance for coronary vasospasm across ablation energy sources and continued evaluation of preventive vasodilator strategies.

## Lead author biography


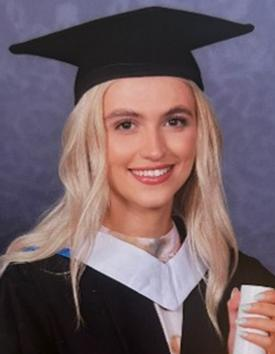
Dr Casey L. Nash is a physician graduated from Cardiff University School of Medicine with Bachelor of Medicine, Bachelor of Surgery with Honors. She is due to complete her foundation training within the Swansea Bay University Health Board in Wales, with the aim of undergoing internal medicine training thereafter.

## Supplementary Material

ytag649_Supplementary_Data

## Data Availability

The de-identified participant data underlying this article are not publicly available in order to protect patient privacy. All data relevant to the case are included in the article and accompanying figures/videos.
